# A Proteomics and Transcriptomics Investigation of the Venom from the Barychelid Spider *Trittame loki* (Brush-Foot Trapdoor)

**DOI:** 10.3390/toxins5122488

**Published:** 2013-12-13

**Authors:** Eivind A.B. Undheim, Kartik Sunagar, Volker Herzig, Laurence Kely, Dolyce H.W. Low, Timothy N.W. Jackson, Alun Jones, Nyoman Kurniawan, Glenn F. King, Syed A. Ali, Agostino Antunes, Tim Ruder, Bryan G. Fry

**Affiliations:** 1 Venom Evolution Lab, School of Biological Sciences, University of Queensland, St. Lucia, Queensland 4072, Australia; E-Mails: eivindandreas@gmail.com (E.A.B.U.); lkely@live.com (L.K.), dol_yce@hotmail.com (D.H.W.L.); tnwjackson@gmail.com (T.N.W.J.); dr.syedabidali@gmail.com (S.A.A.); timruder@yahoo.com.au (T.R.); 2 Institute for Molecular Bioscience, University of Queensland, St. Lucia, Queensland 4072, Australia; E-Mails: v.herzig@imb.uq.edu.au (V.H.); a.jones@imb.uq.edu.au (A.J.); glenn.king@imb.uq.edu.au (G.F.K.); 3 CIMAR/CIIMAR, Centro Interdisciplinar de Investigação Marinha e Ambiental, Universidade do Porto, Rua dos Bragas 177, Porto 4050-123, Portugal; E-Mails: anaturalist@gmail.com (K.S.); aantunes777@gmail.com (A.A.); 4 Departamento de Biologia, Faculdade de Ciências, Universidade do Porto, Rua do Campo Alegre, Porto 4169-007, Portugal; 5 Centre for Advanced Imaging, University of Queensland, St. Lucia, Queensland 4072, Australia; E-Mail: nyoman.kurniawan@cai.uq.edu.au; 6 HEJ Research Institute of Chemistry, International Centre for Chemical and Biological Sciences (ICCBS), University of Karachi, Karachi-75270, Pakistan

**Keywords:** venom, spider, mygalomorph, toxin, evolution

## Abstract

Although known for their potent venom and ability to prey upon both invertebrate and vertebrate species, the Barychelidae spider family has been entirely neglected by toxinologists. In striking contrast, the sister family Theraphosidae (commonly known as tarantulas), which last shared a most recent common ancestor with Barychelidae over 200 million years ago, has received much attention, accounting for 25% of all the described spider toxins while representing only 2% of all spider species. In this study, we evaluated for the first time the venom arsenal of a barychelid spider, *Trittame loki*, using transcriptomic, proteomic, and bioinformatic methods. The venom was revealed to be dominated by extremely diverse inhibitor cystine knot (ICK)/knottin peptides, accounting for 42 of the 46 full-length toxin precursors recovered in the transcriptomic sequencing. In addition to documenting differential rates of evolution adopted by different ICK/knottin toxin lineages, we discovered homologues with completely novel cysteine skeletal architecture. Moreover, acetylcholinesterase and neprilysin were revealed for the first time as part of the spider-venom arsenal and CAP (CRiSP/Allergen/PR-1) were identified for the first time in mygalomorph spider venoms. These results not only highlight the extent of venom diversification in this neglected ancient spider lineage, but also reinforce the idea that unique venomous lineages are rich pools of novel biomolecules that may have significant applied uses as therapeutics and/or insecticides.

## 1. Introduction

In contrast to “modern” spiders (infraorder Araneomorphae), spider families in the infraorder Mygalomorphae have many characteristics that are considered primitive including large paraxial fangs and a complete reliance on book lungs for atmospheric gas exchange [1,2]. The family Barychelidae comprises 44 genera and 307 extant species [3] accounting for 11% of all mygalomorphs, making it one of the most taxonomically diverse mygalomorph spider families. However, in striking contrast with their over 200 million year old sister family Theraphosidae (commonly termed “tarantulas”), which is probably the most thoroughly studied spider family with venom gland transcriptomes of several species already examined [4,5,6,7], barychelids have so far been entirely neglected by toxinological research. Over 200 peptide toxins have been described from theraphosid venoms (accounting for 25% of all described spider toxins while representing only 2% of all spider species) [8], whereas not even a single barychelid venom has been examined to date.

Spider venoms in general are complex mixtures of salts, low molecular mass compounds, acylpolyamines, peptide toxins, and enzymes [9,10]. Primitive spider venoms such as those of theraphosids are dominated by cystine-knotted peptide toxins (“knottins”) with molecular masses of 3–7 kDa that mainly act as modulators of ion channels to provide rapid incapacitation of prey [5,11]. Since they are closely related to theraphosids with a common ancestor dating back to the Triassic [12], one might assume a similar overall composition for barychelid venoms. However, it remains to be examined whether barychelid venoms have evolved novel peptide-toxin scaffolds and/or novel venom components with new modes of action during the 200 million years of independent evolution since their divergence from theraphosids.

Recently, spider-venom peptides have been suggested as leads for the development of novel therapeutics [13] and insecticides [14]. Hence, the identification of novel structural scaffolds from barychelid venoms might add to the pool of already known spider-toxin scaffolds, thereby increasing the chances of selecting useful lead candidates for insecticide and/or drug development. In order to unravel some of these hidden treasures, we have studied the venom gland proteome of the Australian barychelid *Trittame loki* using a combination of transcriptomic, peptidomic and bioinformatic methods. We present the first toxin sequences from barychelid venom, which includes novel cysteine scaffolds, thus providing new insights into the evolution of spider venoms.

## 2. Results and Discussion

**Figure 1:**
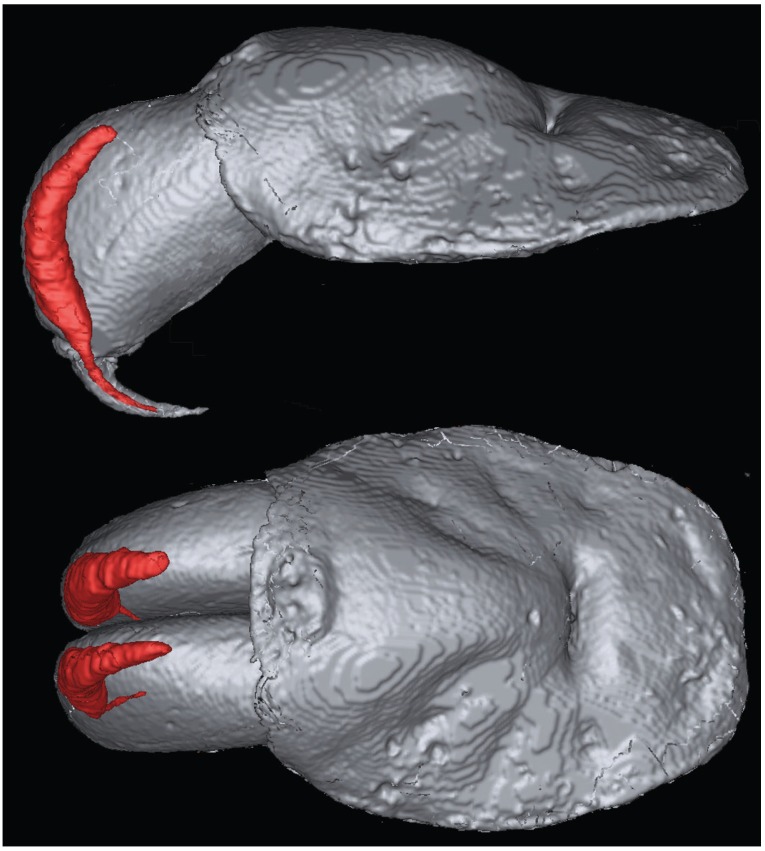
Magnetic resonance imaging of *Trittame loki* venom glands.

**Figure 2:**
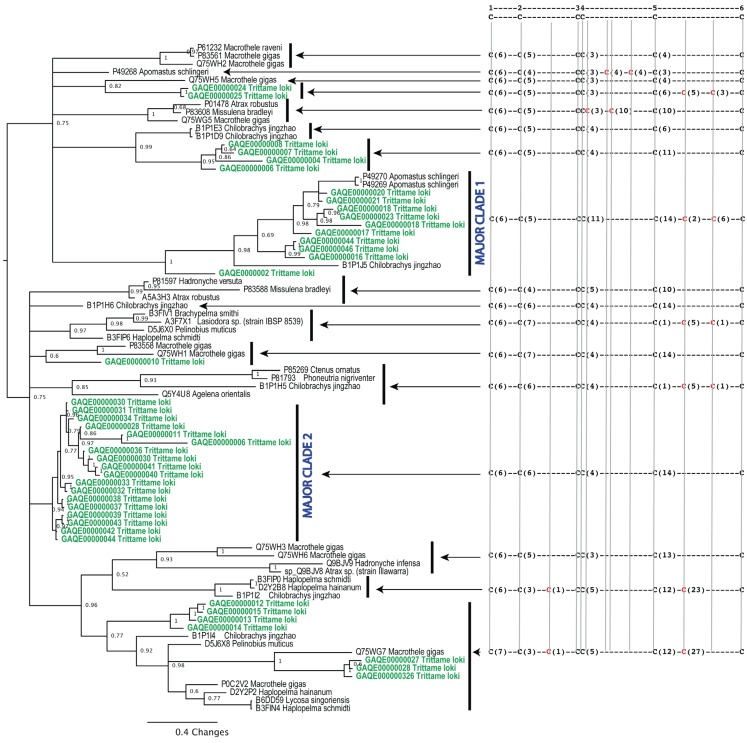
Phylogenetic reconstruction of *Trittame loki* and related inhibitor cystine knot (ICK)/knottin peptide toxins, conserved ancestral cysteines are shown in black, newly evolved cysteines are in red. Sequences obtained in this study are in green. Signal peptides are shown in lowercase.

**Figure 3:**
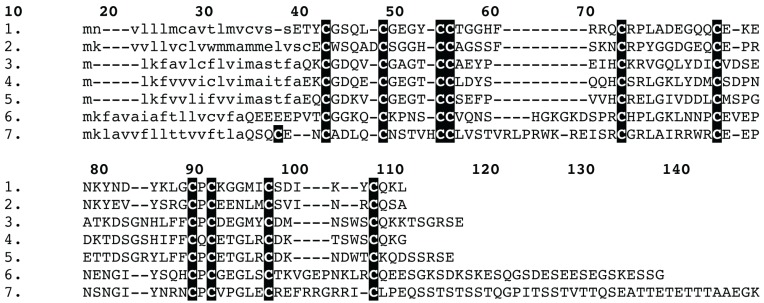
Sequence alignment of spider venom colipase venom peptides: (1) *Trittame loki* COLIPASE-1; (2) D2Y2E5 *Haplopelma hainanum*; (3) Q5D233 *Hadronyche infensa*; (4) Q5D231 *Hadronyche* sp. (strain 20); (5) Q5D232 *Hadronyche* sp. (strain 20); (6) B1P1J0 *Chilobrachys jingzhao*; and (7) B1P1J2 *Chilobrachys jingzhao.* Signal peptides are shown in lowercase.

**Figure 4:**
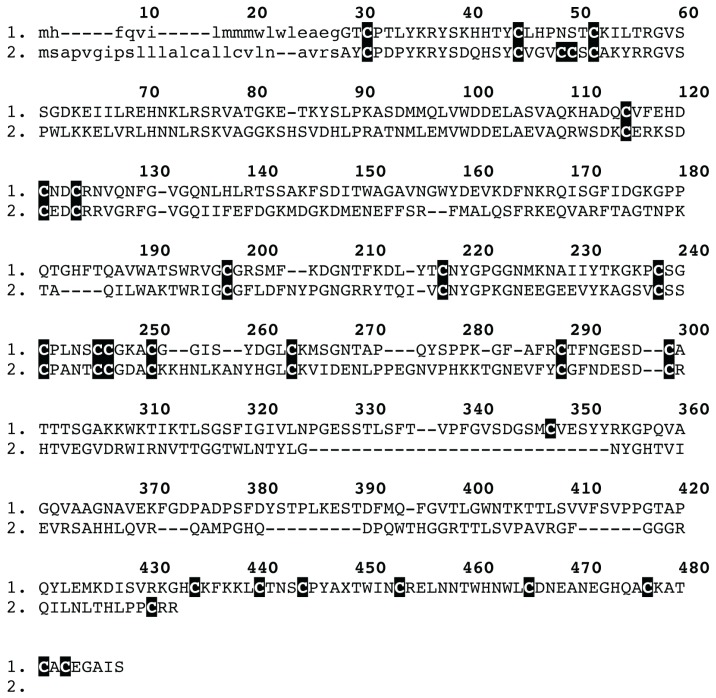
Sequence alignment of spider venom CAP (CRiSP/Allergen/PR-1) venom peptides: (1) *Trittame loki* CAP-1; and (2) A9QQ26 *Lycosa singoriensis.* Signal peptides are shown in lowercase.

**Figure 5:**
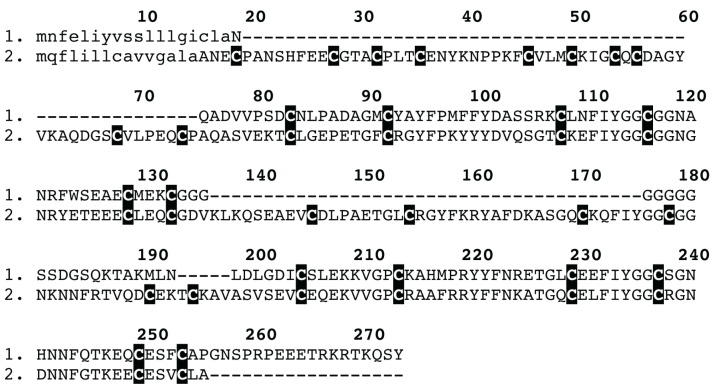
Sequence alignment of spider venom kunitz venom peptides: (1) *Trittame loki* KUNITZ-1; and (2) E7D1N7 *Latrodectus hesperus.* Signal peptides are shown in lowercase.

**Figure 6:**
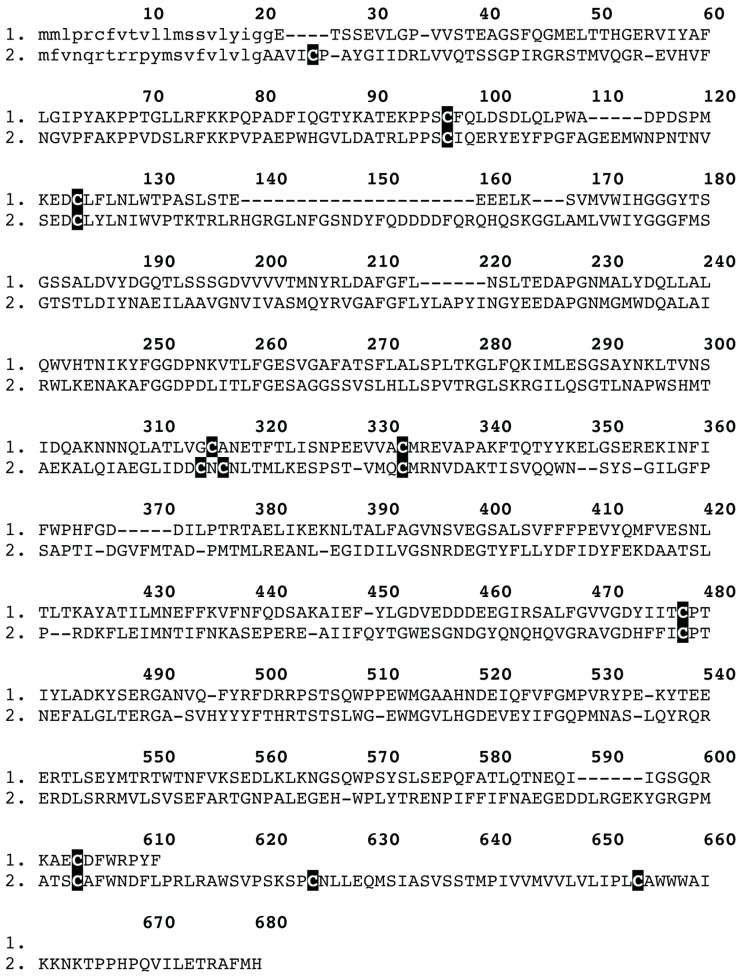
Sequence alignment of the *Trittame loki* venom acetylcholinesterase and the non-venom homologue P56161 *Anopheles stephensi.* Signal peptides are shown in lowercase.

**Figure 7:**
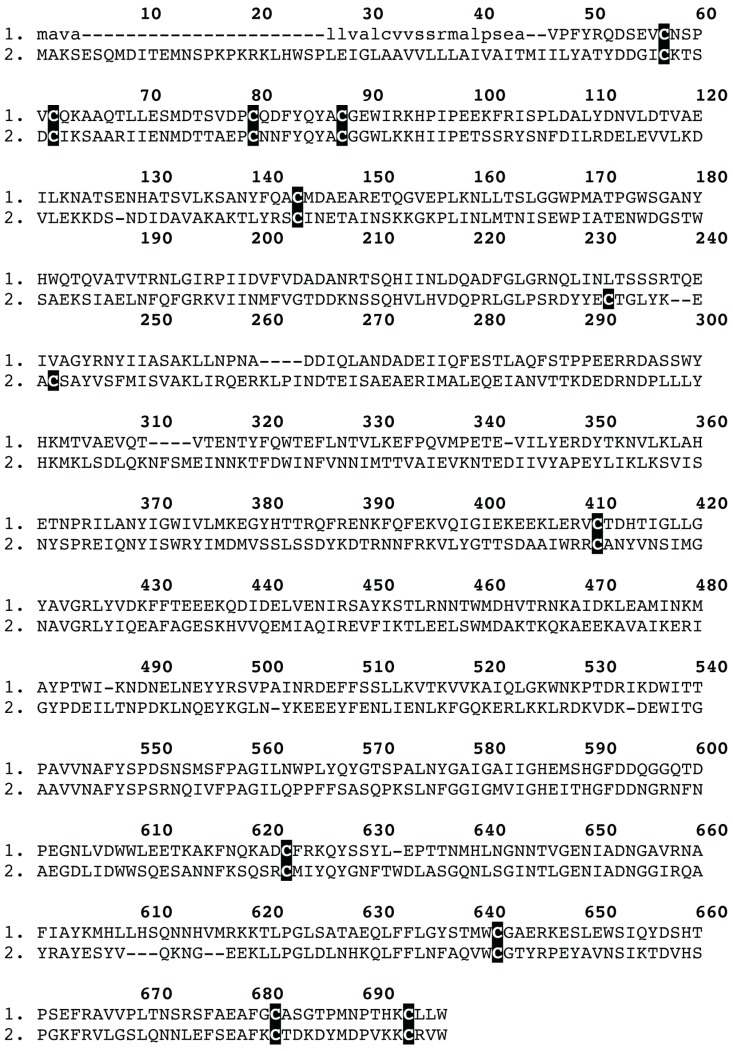
Sequence alignment of the *Trittame loki* venom neprilysin and the snake venom convergent neprilysin homologue T1E4Z0 *Crotalus horridus*. Signal peptides are shown in lowercase.

Magnetic resonance imaging (MRI) revealed that *T. loki* retained the primitive characteristic of large fangs and venom glands contained entirely within the massive chelicerae (Figure 1). Transcriptomics recovered six toxin types: 42 diverse isoforms of the classic ICK/knottin spider-venom peptides (Figure 2); variants of the previously characterised prokineticin family (Figure 3) [15,16], CAP (CRiSP/Allergen/PR-1) (Figure 4) and kunitz (Figure 5) domain proteins, with CAP sequenced for the first time from any mygalomorph spider, and the first discovery of the enzymes acetylcholinesterases (Figure 6) and neprilysin (Figure 7) in spider venom. Proteomics confirmed that each toxin type was translated and present in the secreted venom (Supplementary Table 1), except for kunitz which was not detected by mass spectrometry.

**Table 1:** Molecular evolution analyses of *Trittame loki* major ICK toxin clades.

		SLAC ^a^	FEL ^b^	REL ^c^	MEME ^d^	FUBAR ^e^	Integrative ^f^	BSR ^g^	PAML ^h^	
							SLAC + FEL + REL + MEME		M8	M2a
**Clade I**	ω > 1 ^i^	0	2	22	2 sites	13	22	3	17	12
	ω < 1 ^j^	0	4	0		0	4		(9 + 8)	(6 + 6)
	ω =	1.41	-	1.62		-	-		1.81	1.81
**Clade II**	ω > 1 ^i^	0	0	0	0 sites	0	0	4	5	3
	ω < 1 ^j^	3	11	0		14	15		(1 + 4)	(1 + 2)
	ω =	0.42	-	0.47		-	-		0.67	0.67

**Legend:** ^a^: Single Likelihood Ancestor Counting; ^b^: Fixed-effects likelihood; ^c^: Random-effects likelihood; ^d^: Sites detected as experiencing episodic diversifying selection (0.05 significance) by the Mixed Effects Model Evolution (MEME); ^e^: Fast Unconstrained Bayesian AppRoximation; ^f^: Sites detected in common by SLAC, FEL, REL, MEME and FUBAR (the integrative approach); ^g^: Number of branches detected by the branch-site REL (Random effects likelihood) test as episodically diversifying; ^h^: Positively selected sites detected by the Bayes Empirical Bayes approach implemented in M8 and M2a. Sites detected at 0.99 and 0.95 significance are indicated in the parenthesis; ^i^: Number of positively selected sites at 0.05 significance (for SLAC, FEL) or 50 Bayes factor (for REL); or number of sites under pervasive diversifying selection at 0.9 posterior probability (FUBAR); ^j^: Number of negatively selected sites at 0.05 significance (for SLAC, FEL) or 50 Bayes factor (for REL); or number of sites under pervasive purifying selection at 0.9 posterior probability (FUBAR); ω**:** mean dN/dS.

The most abundant transcript type was of the ICK/knottin neurotoxic peptide type which showed significant evidence of accelerated evolution and diversification. This diversity was accompanied by extensive variation in cysteine pattern including multiple independent *de novo* evolution of novel cysteines (Figure 2). These peptides were phylogenetically diverse, with the *T. loki* ICK/knottin toxins forming eight clades, with two of them being particularly large and divergent (Figure 2). Notably the first of the major clades of ICK peptides also had a pair of newly evolved cysteines, as did less diverse sets. We assessed the molecular evolutionary history of genes encoding these toxins using state-of-the-art selection assessment methods The one-ratio model, the simplest of the lineage-specific models, estimated an ω of 1.36 and 0.46 for the major ICK/knottin toxin clades 1 and 2, respectively (Supplementary Tables 2.1 and 2.2). This highly conservative model can only detect positive selection when the ω ratio, averaged over all sites along the lineages in a phylogenetic tree, is significantly greater than one. Hence, the ω of 1.36 estimated for the major ICK/knottin toxin clade 1 clearly highlights the dominant role of positive selection in shaping their evolutionary history. Since lineage-specific models often fail to detect episodic diversifying selection that only affects certain sites in protein-encoding genes, we further employed site-specific models (Table 1 and Supplementary Tables 2.1 and 2.2). Model 8 estimated an ω of 1.81 and 0.67 for the major ICK/knottin toxin clades 1 and 2, respectively, highlighting the significant influence of positive selection on the evolution of the major ICK/knottin toxin clade 1 (Table 1 and Supplementary Tables 2.1 and 2.2). In contrast to the rapidly evolving clade 1, an ω of 0.67 estimated for the ICK/knottin toxin clade 2 indicated that this toxin clade has evolved under significant constraints of negative selection pressure and that the major part of its coding sequence has remained extremely well conserved over evolutionary time. The Bayes Empirical Bayes (BEB) method identified as many as 17 (33% of sites) positively selected sites in the major ICK/knottin toxin clade 1 and detected only 5 (11% of sites) positively selected sites in toxin clade 2 (Table 1 and Supplementary Tables 2.1 and 2.2), further highlighting the differential influence of evolutionary selection pressures on these two toxin clades. Other methods of evaluating the nature of selection, such as FEL, REL, MEME, FUBAR and integrative approach, conclusively supported these findings and indicated that in contrast to the rapidly evolving clade 1, clade 2 remains extremely well conserved (Table 1).

With a combined nucleotide and amino acid-level approach, we were able to identify seven and five positively selected sites in the major ICK/knottin toxin clades 1 and 2, respectively (Supplementary Table 3). Interestingly, all five hypermutational sites identified in clade 2 were extremely varied in terms of biochemical and/or structural properties, relative to their ancestral residues. Radical mutations are more likely to influence the fitness of the organism, since they are capable of altering the structure and/or function of the protein, in contrast to neutral mutations that result in the introduction of residues with similar biochemical properties. Evolutionary fingerprint analyses clearly depicted several residues in the major ICK/knottin toxin clade 1 as evolving under the influence of positive selection, while a majority of sites in the toxin clade 2 were found to be evolving under the regime of negative-selection (Supplementary Figure 1). The branch-site REL test detected 4 and 3 branches respectively in the two major ICK/knottin toxin clades as evolving under episodic bursts of adaptation (Supplementary Figure 2).

Thus, the evidence provided by various analyses, such as codeml site and lineage-specific models (M8, M2a, M3 and M0), various models of HyPhy (SLAC, FEL, REL, MEME and FUBAR), the amino acid-level approach implemented in TreeSAAP, the evolutionary fingerprint analyses and branch-site REL (Table 1; Supplementary Tables 2.1, 2.2 and 3; and Supplementary Figures 1 and 2), clearly highlighted the differential rate of evolution adopted by the major ICK/knottin toxin clades and is indicative of extreme venom diversification in the barychelid spider *T. loki*. The major ICK/knottin toxin clade 1 in particular was found to have evolved under the significant influence of positive selection, while most codon sites in clade 2 evolved under the regime of negative selection and thus remained well conserved. We speculate that the lack of variation in toxin clade 2 might be the result of selection pressures exerted by their target receptors in the form of steric requirements that influence binding efficiency. The conspicuous rapid accumulation of variations under the influence of positive Darwinian selection in the major ICK/knottin toxin clade 1 is strongly suggestive of its role in prey envenoming. We propose that these toxins are highly likely to play a central role in prey-envenoming and capture, and are likely to be involved in a co-evolutionary predator-prey arms race with their molecular targets in prey animals. A handful of positively selected sites were detected in the coding sequences of ICK/knottin toxin clade 2, indicating that even these highly conserved toxins accumulate variations, probably to fine-tune their biochemical activities. Not-surprisingly, we identified a large number of novel cysteine variants in this rapidly evolving ICK/knottin peptide toxin class (Figure 2), pointing towards potential wide scale neofunctionalisation. Experimental examinations are warranted to precisely understand the stark difference in the evolutionary regimes adopted by the aforementioned toxin clades.

Our identification of enzymes like acetylcholinesterase and neprilysin in the venom of spiders points to a more complex spider-venom arsenal than previously realised. It is noteworthy that, as indicated by the proteomic data (Supplementary table 1), these enzymes are not just present in trace amounts in the venom. This, together with the lack of identification of other proteins with potentially metabolic roles in the venom, suggests that both acetylcholinesterase and neprilysin are likely to play a role in envenomation. While acetylcholinesterase is known for reducing the amount of the neurotransmitter acetylcholine at synapses, neprilysin is a metalloprotease that might be involved in the degradation of extracellular matrix around synapses [9]. Thus, both enzymes might participate in prey envenoming and capture, with neprilysin aiding the action of acetylcholinesterase and neurotoxic peptides by facilitating their access to synaptic targets.

## 3. Experimental Section

### 3.1. Specimens

*Trittame loki* specimens were collected from Cairns, Queensland, with all specimens collected within 100 meters of each other.

### 3.2. Magnetic Resonance Imaging

MRI was used to obtain a three-dimensional (3D) shape of the venom glands without intrusive dissection or sectioning techniques. For fixation, one specimen was anaesthetized by CO_2_ for 30 min and the entire prosoma placed in 4% NBF. Prior to imaging, NBF was removed by four one hour of washing steps in phosphate buffered saline (PBS) and incubated overnight in 0.1% Magnevist^®^ (Bayer, Germany) in PBSÓ. After removal of NBF, the sample was submersed in perfluoro-ether Fomblin (Solvay Solexis, Italy) and placed under vacuum to prevent air artifacts. Imaging was performed on a 16.4 T (700 MHz) vertical 89-mm-bore systems (Bruker BioSpin, Rheinstetten, Germany) using a Bruker Micro 2.5 gradient system (2.5 G/cm A) and transmit/receive radiofrequency coils with diameter of 10 mm quadrature birdcage resonator (M2M Imaging, Brisbane, Australia). Bruker ParaVision 5.0 software was used for image acquisition and anatomical images were acquired using a 3D FLASH (Fast Low Angle Shot) gradient echo sequence. The imaging parameters were: TR/TE = 40/8 ms, flip angle 20°, 4–8 excitations. The field-of-view and matrix sized to fit the sample with the resulting voxels having 30 µm isotropic resolution. Total scan time was 12 h. MRI data was processed using Medical Imaging Processing, Analysis, and Visualization v6.0.0 (MIPAV) and 3D image segmentation, surface rendering and volumetric measurements of the glands were performed manually using ITK-SNAP.

### 3.3. Transcriptome Construction

Paired venom glands were dissected out and pooled from nine mature females on the fourth day after venom depletion by electrostimulation. Total RNA was extracted using the standard TRIzol Plus method (Invitrogen), and extracts were then enriched for mRNA using a RNeasy mRNA mini kit (Qiagen). mRNA was reverse transcribed, fragmented, and ligated to a unique 10-base multiplex identifier (MID) tag prepared using standard protocols and applied to one PicoTitrePlate (PTP) for simultaneous amplification and sequencing on a Roche 454 GS FLX + Titanium platform (Australian Genome Research Facility). Automated grouping and analysis of sample-specific MID reads informatically separated sequences from the other transcriptomes on the plates, which were then post-processed to remove low quality sequences before *de novo* assembly into contiguous sequences (contigs) using v3.4.0.1 of the MIRA software program. Assembled contigs were processed using CLC Main Work Bench (CLC-Bio) and the Blast2GO bioinformatic suite [17,18] to provide Gene Ontology, BLAST and domain/Interpro annotation. The above analyses assisted in rationalisation of the large numbers of assembled contigs into phylogenetic “groups” for detailed phylogenetic analyses as outlined below. Assembly of the data gave 4711 contigs from the 2,340,167 assembled bases, with an average of 497 bases per contig. The raw assembly has been deposited to the NCBI Bioproject repository with the accession number PRJNA189679. For individual sequences analysed in this study, Genbank accession numbers are GAQE00000001-GAQE00000047. It should be noted that only full length sequences were curated, while fragments were not included in the analyses.

### 3.4. Bioinformatics

#### 3.4.1. Phylogenetics

Toxin sequences were identified by comparison of the translated DNA sequences with those of previously characterised toxins using a BLAST search [19] implemented in the UniProtKB protein database. Phylogenetic analyses were conducted not only using the translated amino acid sequences recovered in this study, but also those already published, and the molecular evolutionary history of the *T. loki* ICK peptides was reconstructed. All sequences obtained in this study are indicated by their Genbank accession numbers and sequences from previous studies are referred to by their UniProtKB accession numbers. Resultant sequence sets were aligned using CLC Main Work Bench, using the default algorithm. Sequence alignments are depicted with their signal peptide shown in lowercase and cysteines highlighted in bold. Datasets were analysed using a Bayesian inference implemented in MrBayes version 3.2.1 [20] and the command block lset rates = gamma with prset aamodelpr = mixed, which enables the program to optimize between nine different amino acid substitution matrices, was employed. The analysis was performed by running a minimum of 1 × 10^7^ generations in four chains, and saving every 100th tree. The log-likelihood score of each saved tree was plotted against the number of generations to establish the point at which the log likelihood scores reached their asymptote, and the posterior probabilities for clades established by constructing a majority-rule consensus tree for all trees generated after completion of the burn-in phase. Supplementary File 2 contains alignment details.

#### 3.4.2. Test for Recombination

Since phylogenetic and evolutionary interpretations [21] are influenced by recombination, we employed Single Breakpoint algorithm implemented in the HyPhy package and assessed the role of recombination on ICK peptide-encoding genes examined in this study [22,23]. When potential breakpoints were detected using the small sample Akaike information Criterion (AICc), the sequences were compartmentalized before conducting selection analyses.

#### 3.4.3. Selection Analyses

We evaluated the influence of natural selection on the venom of the barychelid spider *T. loki* using maximum-likelihood models [24,25] implemented in CODEML of the PAML package [26]. We initially employed the one-ratio model that assumes a single ω for the entire phylogenetic tree. However, this model tends to be very conservative and can only detect positive selection when the ω ratio averaged over all the sites along the lineage is significantly greater than one. Since a lineage-specific model like the one-ratio model assumes a single ω for the entire tree, it often fails to identify regions in proteins that might be affected by episodic selection pressures and hence underestimates the strength of selection. We therefore employed site-specific models that estimate positive selection statistically as a non-synonymous-to-synonymous nucleotide-substitution rate ratio (ω) significantly greater than 1. We compared likelihood values for three pairs of models with different assumed ω distributions as no *a priori* expectation exists for the same: M0 (constant ω rates across all sites) *versus* M3 (allows ω to vary across sites within “n” discrete categories, *n* ≥ 3); M1a (a model of neutral evolution) where all sites are assumed to be either under negative (ω < 1) or neutral selection (ω = 1) *versus* M2a (a model of positive selection) which in addition to the site classes mentioned for M1a, assumes a third category of sites; sites with ω > 1 (positive selection) and M7 (Beta) *versus* M8 (Beta and ω), and models that mirror the evolutionary constraints of M1 and M2 but assume that ω values are drawn from a beta distribution [27]. Only if the alternative models (M3, M2a and M8: allow sites with ω > 1) show a better fit in Likelihood Ratio Test (LRT) relative to their null models (M0, M1a and M7: do not allow sites with ω > 1), are their results considered significant. LRT is estimated as twice the difference in maximum likelihood values between nested models and compared with the χ^2^ distribution with the appropriate degree of freedom—the difference in the number of parameters between the two models. The Bayes empirical Bayes (BEB) approach [28] was used to identify amino acids under positive selection by calculating the posterior probabilities that a particular amino acid belongs to a given selection class (neutral, conserved or highly variable). Sites with greater posterior probability (*PP* ≥ 95%) of belonging to the “ω > 1 class” were inferred to be positively selected.

Single Likelihood Ancestor Counting (SLAC), Fixed-Effects Likelihood (FEL), and Random Effects Likelihood (REL) models [29] implemented in HyPhy [22] were employed to provide additional support to the aforementioned analyses and to detect sites evolving under the influence of positive and negative selection. Mixed Effects Model Evolution (MEME) [30] was also used to detect episodic diversifying selection. A complementary protein-level approach implemented in TreeSAAP [31] was used to assess the radicalness of mutations. The proportion of sites under different regimes of selection was depicted using an evolutionary fingerprint analysis, which uses an ESD algorithm, implemented in datamonkey [32]. We further employed branch-site REL to identify lineages undergoing episodic bursts of adaptation [33].

### 3.5. Proteomics

Specimens were anaesthetized using carbon dioxide gas. Polyethylene equipment was used to collect and process samples in all cases. Samples were subsequently filtered using 20 Å syringe filters to remove large mucoidal strands and then lyophilised. Reduction and alkylation was undertaken by redissolving 3 µg of sample in 50 µL of 100 mM ammonium carbonate. 50 µL of 2% iodoethanol/0.5% triethylphosphine in acetonitrile was then added to the re-dissolved sample. The reaction mixture was incubated for 2 h at 37 °C, before being dried by vacuum centrifugation and re-suspended in 20 µL of 2.5% acetonitrile (ACN), 1% formic acid. Additionally, 3 µg of reduced and alkylated sample was resuspended in 20 µL of 40 mM ammonium bicarbonate, before being incubated overnight with 750 ng trypsin. Digestion was stopped by addition of 1 µL of concentrated formic acid. 5 µL (0.75 µg) of each sample was processed by LC/MS using a Vydac Everest C_18_ column (150 mm × 7.5 mm, 5 mm particle size, 300 Å pore size) at a flow of 0.5 mL/min and a gradient of 1%–40% solvent B (90% ACN, 0.1% formic acid) over 25 min coupled with an AB SCIEX 5600 Triple TOF mass spectrometer. MS^2^ spectra were acquired at a rate of 20 scans/second. MS^2^ spectra were searched against the translated cDNA library using Proteinpilot v4.0 (ABSciex) and further analysed using CLC Main Workbench v6.6.

## 4. Conclusions

In conclusion, our multidisciplinary approach has revealed: (i) the differential rate of evolution adopted by *T. loki* toxins: some clades are extremely well conserved under the influence of negative selection, likely as a result of the steric requirements that influence binding efficiencies, while others rapidly accumulate variations under positive selection; (ii) the evolution of novel molecular scaffolds through the acquisition of new cysteine residues; (iii) the important role of positive selection in the evolution and diversification of ICK/knottin toxin clades; (iv) the presence of novel enzymes (acetylcholinesterase and neprilysin) for the first time in spider venom; and (v) the presence of CAP for the first time in the mygalomorph spider venom. Venom components employed for predation often experience a greater influence from positive selection and accumulate variations that are not only necessary for acquiring different types of prey [34,35,36], but also in escaping host immune responses [37]. The likelihood of identifying novel venom components for drug design is theoretically higher in organisms with extremely variant and rapidly evolving venom arsenals. The results of this study reinforce the significance of studying even obscure spider lineages. These results also add to the growing body of knowledge regarding structural convergence of venom components as both acetylcholinesterase and neprilysin have been recruited into the venoms of other animals [36,37].

## Supplementary Files

Supplementary File 1 Supplementary (ZIP, 313 KB)
