# Supplementary material for: A Proteomics and Transcriptomics Investigation of the Venom from the Barychelid Spider Trittame loki (Brush-Foot Trapdoor)
Source: Toxins (Basel). 2013 Dec 13;5(12):2488–503. doi: 10.3390/toxins5122488 (PMC3873697; doi:10.3390/toxins5122488)
Supplement: Supplementary File 1 — Supplementary (ZIP, 313 KB) [file toxins-05-02488-s001.zip › Supplementary material/Supplementary Figure 1 - Evolutionary Fingerprint.pdf]

Supplementary Figure 1. Evolutionary fingerprint of *Trittame loki* major ICK toxin clades

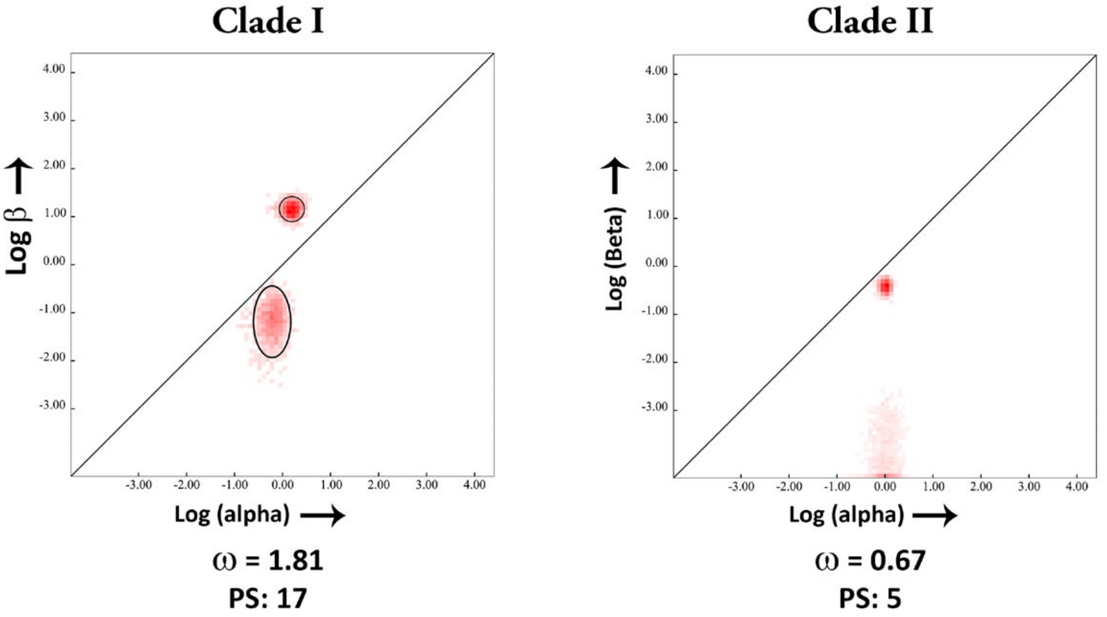

Evolutionary Fingerprint: Estimates of the distribution of synonymous (α) and non-synonymous (β) substitution rates inferred for *Trittame loki* major ICK toxin clades are shown here. The ellipses reflect a Gaussian-approximated variance in each individual rate estimate, and coloured pixels show the density of the posterior sample of the distribution for a given rate. **The diagonal line represents the idealized neutral evolution regime (ω=1), points above and below the line correspond to positive selection (ω>1) and negative selection (ω<1), respectively.** Site model 8 omega (w) along with the total number of positively selected sites detected by its Bayes Empirical Bayes (BEB) approach are also indicated below.
