# Supplementary material for: A Proteomics and Transcriptomics Investigation of the Venom from the Barychelid Spider Trittame loki (Brush-Foot Trapdoor)
Source: Toxins (Basel). 2013 Dec 13;5(12):2488–503. doi: 10.3390/toxins5122488 (PMC3873697; doi:10.3390/toxins5122488)
Supplement: Supplementary File 1 — Supplementary (ZIP, 313 KB) [file toxins-05-02488-s001.zip › Supplementary material/Supplementary Figure 2 - Branch-site REL.pdf]

Supplementary Figure 2. **Branch-site REL of *Trittame loki* major ICK toxin clades**

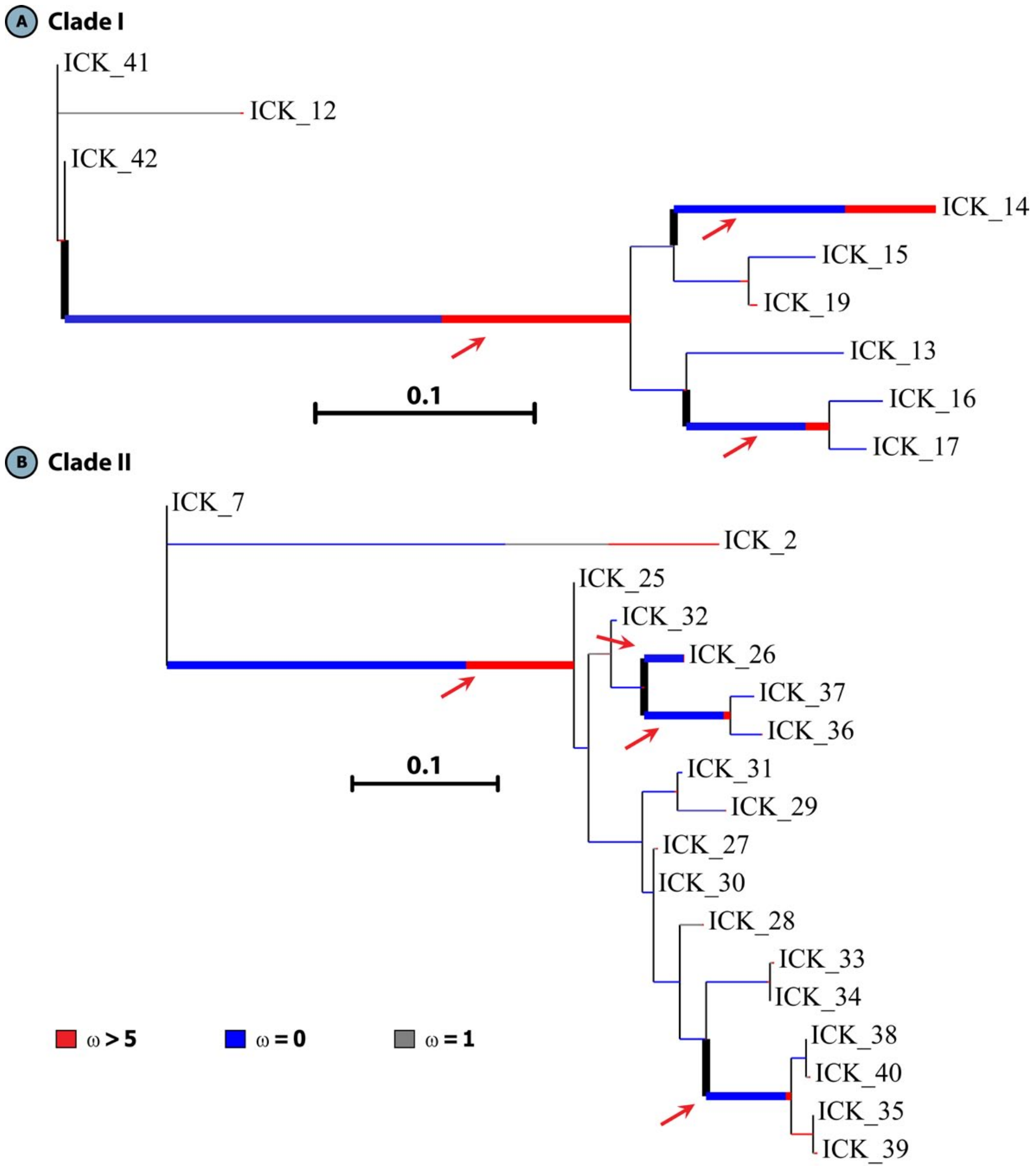

Branch-site REL: The hue of each colour indicates strength of selection, with primary red corresponding to ω > 5, primary blue to ω=0 and grey to ω=1. The width of each colour component represents the proportion of sites in the corresponding class. Thicker branches have been classified as undergoing episodic diversifying selection (indicated by arrows) by the sequential likelihood ratio test at corrected p ≤ 0.05
