# Supplementary material for: A Proteomics and Transcriptomics Investigation of the Venom from the Barychelid Spider Trittame loki (Brush-Foot Trapdoor)
Source: Toxins (Basel). 2013 Dec 13;5(12):2488–503. doi: 10.3390/toxins5122488 (PMC3873697; doi:10.3390/toxins5122488)
Supplement: Supplementary File 1 — Supplementary (ZIP, 313 KB) [file toxins-05-02488-s001.zip › Supplementary material/Supplementary Table 2.1 and 2.2 - Maximum-likelihood parameter estimates for ICK-knottin toxin clades.docx]

**Supplementary Table 2.1** Maximum-likelihood parameter estimates for ICK/knottin toxin clade 1

| Model | Likelihood () | ω_0_^a^ | Parameters | Sign.^b^ | No. of Sites with ω > 1^c^ |
| --- | --- | --- | --- | --- | --- |
|  |  |  |  |  | **B.E.B** |
|  |  |  |  |  |  |
| M0 (One ratio) | -1236.493618 | 1.36 | = ω_0_ |  | - |
| M1 (Neutral) | -1218.190148 | 0.68 | P_0_: 0.317  ω_0_: 0.005  P_1_: 0.682  ω_1_:1.0 |  | - |
| M2 (Selection)* | -1202.728743 | 1.81 | P_0_: 0.262  ω_0_: 0.0  P_1_: 0.407  ω_1_:1.0  P_2_: 0.330  ω_2_: 4.27 | P << 0.001 | 6 (PP ≥ 0.99)  6 (P ≥ 0.95) |
| M3 (Discrete)* | -1202.711196 | 1.84 | P_0_: 0.271  ω_0_: 0.0  P_1_: 0.414  ω_1_: 1.09  P_2_: 0.313  ω_2_: 4.43 | P << 0.001 | - |
| M7 (beta) | -1218.215423 | 0.70 | p: 0.01185  q: 0.00500 |  | - |
| M8 (beta and ω)* | -1202.731675 | 1.81 | p_0_: 0.667  p: 0.007  q: 0.005  p1: 0.332  ω: 4.24 | P << 0.001 | 9 (PP ≥ 0.99)  8 (P > 0.95) |

**Legend:**

**a:** dn/ds (weighted average)

**b:** Significance of the model in comparison with the null model

**c:** Number of sites with ω > 1 under the Bayes empirical Bayes approach with a posterior probability (PP) more than or equal to 0.99 and 0.95

***** Models which allow ω > 1

**P > 0.05^N.S^:** Not significant at 0.05

**Supplementary Table 2.2** Maximum-likelihood parameter estimates for ICK/knottin toxin clade 2

| Model | Likelihood () | ω_0_^a^ | Parameters | Sign.^b^ | No. of Sites with ω > 1^c^ |
| --- | --- | --- | --- | --- | --- |
|  |  |  |  |  | **B.E.B** |
|  |  |  |  |  |  |
| M0 (One ratio) | -1851.619506 | 0.46 | = ω_0_ |  | - |
| M1 (Neutral) | -1810.776529 | 0.38 | P_0_: 0.721  ω_0_: 0.153  P_1_: 0.278  ω_1_: 1.0 |  | - |
| M2 (Selection)* | -1797.330128 | 0.67 | P_0_: 0.691  ω_0_: 0.18  P_1_: 0.245  ω_1_:1.0  P_2_: 0.063  ω_2_: 4.75 | P << 0.001 | 1 (PP ≥ 0.99)  2 (P ≥ 0.95) |
| M3 (Discrete)* | -1796.015810 | 0.65 | P_0_: 0.211  ω_0_: 0.0  P_1_: 0.649  ω_1_: 0.37  P_2_: 0.138  ω_2_: 3.007 | P << 0.001 | - |
| M7 (beta) | -1811.902526 | 0.41 | p: 0.39322  q: 0.56216 |  | - |
| M8 (beta and ω)* | -1796.587858 | 2.25 | p_0_: 0.892  p: 0.769  q: 1.545  p1: 0.107  ω: 3.49 | P << 0.001 | 1 (PP ≥ 0.99)  4 (P > 0.95) |

**Legend:**

**a:** dn/ds (weighted average)

**b:** Significance of the model in comparison with the null model

**c:** Number of sites with ω > 1 under the Bayes empirical Bayes approach with a posterior probability (PP) more than or equal to 0.99 and 0.95

***** Models which allow ω > 1
