# Supplementary material for: A Proteomics and Transcriptomics Investigation of the Venom from the Barychelid Spider Trittame loki (Brush-Foot Trapdoor)
Source: Toxins (Basel). 2013 Dec 13;5(12):2488–503. doi: 10.3390/toxins5122488 (PMC3873697; doi:10.3390/toxins5122488)
Supplement: Supplementary File 1 — Supplementary (ZIP, 313 KB) [file toxins-05-02488-s001.zip › Supplementary material/Supplementary Table 3.docx]

Supplementary Table 3. Nucleotide and amino acid-level selection assessment

| **Site** | | **CodeML** | | **TreeSAAP** | |
| --- | --- | --- | --- | --- | --- |
| **Codon** | **AA** | **M2a^a^** | **M8^b^** | **Property^c^** | **Magnitude^d^** |
| **Clade 1** | | | | | |
| 22 | N | 4.173±0.768 | 3.926±0.637 | *−* | *−* |
|  |  | 0.990* | 0.996** |  |  |
| 24 | H | 4.19±0.736 | 3.933±0.619 | *−* | *−* |
|  |  | 0.995** | 0.998** |  |  |
| 26 | A | 4.104±0.879 | 3.898±0.692 | *−* | *−* |
|  |  | 0.970* | 0.988* |  |  |
| 31 | R | 4.189±0.738 | 3.933±0.62 | *−* | *−* |
|  |  | 0.995** | 0.998** |  |  |
| 52 | G | 4.178±0.759 | 3.929±0.628 | *−* | *−* |
|  |  | 0.991** | 0.997** |  |  |
| 56 | M | 4.068±0.928 | 3.885±0.717 | *E_l_* | 8 |
|  |  | 0.960* | 0.984* |  |  |
| 58 | V | 3.944±1.073 | 3.828±0.812 | *E_l_* | 8 |
|  |  | 0.925 | 0.967* |  |  |
| 61 | D | 3.888±1.134 | 3.796±0.862 | *E_l_* | 8 |
|  |  | 0.908 | 0.957* |  |  |
| 65 | Y | 4.194±0.727 | 3.935±0.616 | *E_l_* | 8 |
|  |  | 0.996** | 0.999** |  |  |
| 69 | R | 4.161±0.79 | 3.921±0.647 | *−* | *−* |
|  |  | 0.986* | 0.994** |  |  |
| 74 | S | 4.144±0.82 | 3.913±0.664 | *−* | *−* |
|  |  | 0.981* | 0.992** |  |  |
| 75 | E | 4.203±0.709 | 3.938±0.609 | *−* | *−* |
|  |  | 0.999** | 1.000** |  |  |
| 77 | S | 3.820±1.186 | 3.771±0.892 | *−* | *−* |
|  |  | 0.891 | 0.950* |  |  |
| 78 | K | 4.116±0.862 | 3.904±0.681 | *−* | *−* |
|  |  | 0.974* | 0.989* |  |  |
| 81 | - | 3.941±1.092 | 3.804±0.857 | *E_l_* | 8 |
|  |  | 0.922 | 0.959* |  |  |
| 83 | F | 4.191±0.733 | 3.933±0.619 | *E_l_* | 8 |
|  |  | 0.995** | 0.998** |  |  |
| 87 | R | 3.926±1.095 | 3.817±0.83 | *E_l_* | 8 |
|  |  | 0.919 | 0.963* |  |  |
| **Clade 2** | | | | | |
| 76 | N | 4.919±1.842 | 4.280±1.366 | *B_l_, h, α_n_* | 6, 6, 6 |
|  |  | 0.927 | 0.986* |  |  |
| 84 | S | 5.094±1.796 | 4.275±1.394 | *B_l_, h, α_n_, E_SM_* | 6, 6, 6, 6 |
|  |  | 0.952* | 0.981* |  |  |
| 87 | S | 4.743±2.013 | 4.193±1.455 | *B_l_, h, E_SM_* | 6, 6, 6 |
|  |  | 0.880 | 0.962* |  |  |
| 91 | S | 5.072±1.791 | 4.284±1.378 | *B_l_, E_SM_* | 6, 6 |
|  |  | 0.950* | 0.985* |  |  |
| 112 | I | 5.296±1.580 | 4.339±1.323 | *h, E_SM_* | 8, 6 |
|  |  | 1.000** | 1.000** |  |  |
|  | | | | | |

**Amino acid property symbols:** Bulkiness *(B_l_);* Hydropathy *(h);* Long-range non-bound energy *(E_l_);* Power to be at the N-terminus of an alpha helix *(α_n_);* Short- & medium-range non-bound energy *(E_SM_)*

**Legend:**

a: M2a Bayes Empirical Bayes (BEB) posterior probability and post-mean omega indicated in brackets

b: M8 Bayes Empirical Bayes (BEB) posterior probability and post-mean omega indicated in brackets

c: amino acid property under selection

d: magnitude of selection on the amino acid property
